# Public Trust and Policy Compliance during the COVID-19 Pandemic: The Role of Professional Trust

**DOI:** 10.3390/healthcare9020151

**Published:** 2021-02-02

**Authors:** Orachorn Saechang, Jianxing Yu, Yong Li

**Affiliations:** 1 School of Public Affairs, Zhejiang University, 866 Yuhangtang Rd., Hangzhou 310058, China; orachorn.s@zju.edu.cn (O.S.); yujianxing@zju.edu.cn (J.Y.); 2 Zhejiang Gongshang University, No. 18, Xuezheng Str, Hangzhou 310018, China; 3 School of Marxism, Shanghai Maritime University, 1550 Haigang Avenue, Pudong New Area, Shanghai 201306, China

**Keywords:** COVID-19, public trust, policy compliance, public health, personal protective measures

## Abstract

Previous research has shown that public trust is vital for promoting policy compliance, particularly in times of crisis. However, the literature supporting this notion remains scarce, especially in countries which have successfully contained a pandemic despite showing a generally low level of public trust in the government. To address this topic, we conducted a cross-sectional study between February and March of 2020 to examine the relationship between public trust and the compliance of the general public in adopting personal protective measures introduced during the early phase of the coronavirus disease 2019 (COVID-19) outbreak in Thailand. We report our result from a hierarchical regression. We find a positive and significant relationship between public trust in the government and the likelihood of respondents adopting these precautions, more importantly, this relationship was fully mediated by the professional trust as the effect of public trust in the government on policy compliance was reduced by 0.118, namely from (β = 0.133, *p* < 0.001) to (β = 0.015, *p* > 0.05). Married respondents residing in the capital city, with a higher degree of worry were also more likely to comply with these safety measures. In conclusion, the finding sheds light on the dynamic relationship between public trust and policy compliance and offers some implications in times of a global health crisis.

## 1. Introduction

Previous studies indicated that public trust was an important determinant for citizens to comply with public policy and intervention, particularly in times of crisis. Many scholars maintain that pubic trust in the government fosters ordinary citizens to comply with government policy [1,2,3,4,5,6,7]. When the government was perceived as trustworthy, citizens were likely to comply with the regulations [3,5,6]. Moreover, empirical study also suggested that political trust promoted policy compliance and eased policy implementation [8,9]. More importantly, trust in the government was also conducive for the citizens to comply with policy programs voluntarily [4]. On the other hand, a low level of trust in the government could reduce support for government policy [7]. It could also undermine the legitimacy of policy as well as its implementation and lead to a lower level of compliance [10]. In times of uncertainty, trust could be explained as a factor coming from the salient values and past performance which generated confidence and finally led to cooperation [11]. This cooperation would then finally lead to successful policy implementation and result in satisfactory outcomes. However, during the coronavirus disease 2019 (COVID-19) outbreak, we have witnessed that countries with high and low public trust alike responded effectively to the prevention and control of the epidemic. Thus, this calls for a re-examination of the relationship between public trust and its effect on policy compliance and implementation.

Traditionally, a high level of public trust in the government has significantly influenced citizens to comply with policy intervention. As found in the H1N1 pandemic, a high level of trust in the government led to the increasing likelihood of citizens adopting protective measures [12,13]. Despite not knowing much about the nature of the infectious disease, but showing a high level of trust in the government, the majority of Singaporeans followed the control measures, and ultimately, the SARS epidemic has been well controlled and the public felt very satisfied with the government response to the SARS outbreak [14]. If the government managed the crisis properly, their trust could be increased while ineffective management could further deteriorate trust [15]. On the contrary, low trust in the government led to less compliance. During the Ebola outbreak in West Africa, several studies found the mistrust in the government was associated with the likelihood of citizens to comply less with the government control interventions [16,17,18]. If trust was not properly generated, it could lead to a vicious cycle of not complying, hardship, and again back to distrust [16]. This raises alarming concern in a country with low trust in the government of how to increase the likelihood of people complying with the policy intervention in times of crises which finally lead to the success of the epidemic control.

Literature suggested that professional trust was regarded as another crucial determinant for policy compliance. Professional trust can be defined by the willingness of a person to rely on the skills and abilities of experts [19]. It has been found that health agencies enhance public trust and positively influence people’s willingness to adopt recommended behavior [20]. Despite legitimacy and efficacy challenges, vaccination hesitancy could be improved with a higher level of professional trust. Strong recommendations and a higher level of trust placed in professional healthcare workers were associated with a higher acceptance of the vaccine [21,22,23,24,25]. It has been found that parents with higher professional trust are also more likely to have their children vaccinated [24]. By contrast, negative advice from professional healthcare workers was associated with parents’ vaccine hesitancy [25]. Likewise, in responding to uncertainty, the tool of information and the involvement of professionals was found to lead to a successful implementation of policy intervention programs [26,27,28]. However, little is known about how professional trust, in parallel with public trust in the government, impact policy compliance in times of a public health crisis. Thus, there is a need to reassess the relationship between professional trust and the willingness of citizens to comply with the policy intervention measures.

The recent SARS-CoV-2 outbreak has rapidly spread and affected millions of people worldwide since the first case was reported on 31 December 2019 in Wuhan, China. On 11 March, the World Health Organization (WHO) declared COVID-19 as a pandemic when there were 125,260 globally confirmed cases, and about one-third of these cases were found outside China in 117 countries and territories. By late March, the numbers of confirmed cases increased seven-fold and the graph was rapidly rising [29]. At the end of 2020, there were over 83 million laboratory-confirmed cases and nearly 2 million deaths globally in 218 countries and territories, contributing to a fatality rate of 2% [30]. The US alone accounted for nearly a quarter of global confirmed cases and deaths. American regions were especially hard hit, with half of the global accumulated cases. The situation is highly worrying as new daily confirmed cases are still accelerating at the rate of hundreds of thousands globally [29]. This raises significant concern on how to effectively respond to the pandemic in time, especially in developing countries that have now become the epicenter of the outbreak.

Comparatively, the situation in Thailand is considerably contained. Locally transmitted cases were zero for a consecutive seven months from May to November and daily confirmed cases were solely from repatriated people [31]. These returnees were placed under the quarantine system of State Quarantine, Local Quarantine, or Alternative State Quarantine and must be tested twice for SARS-CoV-2 during the 14-day quarantine [32]. In fact, Thailand was the first country outside China to report the laboratory-confirmed case of the COVID-19 on 13 January 2020 [29]. On 15 March, the total confirmed cases in Thailand exceeded 100 before the daily new confirmed cases reached a peak of 188 on 22 March. On 26 March 2020, the government enacted the National Emergency Decree to restrict travel and movement. Four months after the first case and two months after the implementation of the lockdown policy, Thailand reported the first zero daily confirmed cases. By the end of November, there were 3998 confirmed cases, 60 deaths, and 3803 recoveries, contributing to a 1.5% fatality rate and a 95% of recovery rate, respectively [31].

How did Thailand manage to survive such an outbreak during the early phase of the pandemic? The answer is complex. Apart from medical efforts in testing, contact tracing, and quarantine, compliance with policy control measures from the general public to prevent and safeguard collective safety is of utmost significance. Since the COVID-19 is a preventable respiratory disease, it is crucial for the public to adopt personal protective measures as a means to mitigate the spread of the pandemic when the vaccine is not yet available. Trust and compliance have led to effective containment of the pandemic while lower trust led to less compliance in responding to the COVID-19 [33,34,35,36]. However, different socioeconomic status in different regions results in different levels of compliance [33,34,37]. Hence, it is necessary to assess the level of the general public adhering to these safety guidelines from the Asian perspective. More importantly, although most existing studies have emphasized the role of public trust in the prevention and control of public health emergencies, we have seen both traditionally broad public trust countries such as China and Singapore, and others with lower trust in the government like Thailand, effectively responding to the pandemic and achieving decent outcomes in containing the outbreak. Therefore, this raises the question of what is the dynamic relationship between public trust and the prevention and control of the pandemic. At the individual level the question we intend to answer is: How does public trust impact the adoption of personal protective measures? We propose that professional trust could be an alternative approach in mediating the relationship between public trust in the government and policy compliance. In essence, this study aims to assess the compliance of policy control measures from the perspective of public trust. Consequently, the study will not only help to deepen our understanding of the role of public trust during public health crises but also makes an important contribution by offering an experience of a COVID-19 prevention and control strategy.

## 2. Materials and Methods

### 2.1. Study Design and Data Collection

This was a cross-sectional study conducted between 15 February and 25 March of 2020 during the early phase of the COVID-19 pandemic in Thailand. For safety concerns, we distributed questionnaire surveys both online and offline to the general public in Thailand using a snowballing technique. Snowball sampling relied on the first group of targeted members to refer to other members [38]. Recruitment of respondents was completed via social media platforms using existing personal and social contacts. The snowball technique was chosen because at the time of the data collection, the general public started to be aware of the COVID-19 outbreak and it was not convenient to come in contact with a large population due to safety concerns, especially in March when the cases started to soar [39]. Inclusion criteria were individuals who resided in Thailand. Exclusion criteria were illiterate individuals since the survey was self-administered. In addition, we made every effort to include a large sample of 809 respondents. The sample characteristic is quite representative, gender and geographical measure of Bangkok and non-Bangkok were distributed nearly equally. During these five weeks, we received 453 complete online questionnaires. From 500 copies circulated, 382 sets were returned of which 356 were complete, corresponding to 71.2% of the complete responding rate. Finally, a sample size of 809 was used for this study.

### 2.2. Variable Construction and Measures

Variables in this study are divided into three categories and all are recorded using a 5-point Likert scale. Since a Likert scale is widely used in measuring psychological evaluation of trust, we used an indicator 1 for “strongly disagree” to 5 for “strongly agree” when asked the respondents to respond to the corresponding statement in measuring public trust in the government and professional trust [12,16,33]. To measure trust in the government, we asked how much trust the respondents have toward the central government, the local government, and the Ministry of Public Health as well as how much they believe in the capacity of the government to effectively cope with the COVID-19 in terms of the policy, ability to handle the crisis, screening, and treating services. To measure professional trust, we asked how much the respondents trust physicians, dentists, pharmacists, nurses, and their ability to cope with the pandemic effectively.

To measure the adoption of personal protective measures, the respondents were asked about their frequency in practicing six types of safety measure, an indicator of 1 was taken for “never” and 5 for “always”. The recommended safety measures included washing hands with alcohol-based hand rub and avoiding going to crowded places as recommended by the WHO, as well as wearing face masks and using common eating utensils as recommended by Thai authorities [29]. To clarify, eating with common utensils refers to the practice where everyone at the table having his/her own utensils and specific common utensils used for shared dishes in order to eat more hygienically. In addition, we also asked two more questions of wearing the N95 and gargling with salt water.

We used seven control variables including indicators for sex (1 = male, 0 = female); age (<18, 18–29, 30–39, 40–59, 60 and above); marital status (1 = married, 0 = non-married); residential area (1 = Bangkok, 0 = non-Bangkok); education (1 = college degree or above, 0 = below college degree); monthly personal income in THB (<5000, 5000–15,000, 15,001–30,000, 30,001–50,000, <50,000) where 13,803 THB was an average wage in 2016 [40]; and a degree of worry towards the COVID-19 as perceived by the respondents (rating 1 for “not at all” and 5 for “highly worried”).

### 2.3. Statistical Analyses

We employed a statistical tool of IBM SPSS version 23 (IBM, Armonk, NY, USA) and the PROCESS procedure version 3.5 in SPSS [41]. We reported our results from the ordinary least squares (OLS) hierarchical regressions. We further tested the mediating variable effect of professional trust in a relationship between public trust in the government and the policy compliance in adopting personal protective measures from executing 5000 samples of bootstrapping technique at the confidence intervals at 95% using the PROCESS procedure in SPSS [42].

## 3. Results

### 3.1. Sample Characteristics

A total of 809 valid responses were recorded in this study. Table 1 provides a demographic characteristic of the respondents. This is a group of the working-class, nearly half of them aged from 30–39, the majority female, single, and living in Bangkok, the capital city of Thailand. The majority have obtained at least a college degree and earned a middle income in their monthly salary. This group is potentially a target group of people who have a high risk of being infected, as 37 was the average age of the infected cases found in Thailand and half of them resided in Bangkok [31]. Despite a comparatively lower level of respondents being socially or economically affected by the coronavirus (51.1%), 73.1% of them felt worried and anxious.

### 3.2. Characteristics of Study Variables 

Table 2 displays descriptive statistics for the three categories of variables used in this study. Cronbach’s α value were all above 0.7, suggesting that the variables were reliable [43]. Distrust in the government was prevalent among our respondents. Only 21% of respondents expressed their trust toward the central government and with an even lower rate of 16% trusted their local government. However, nearly half of respondents expressed their trust in the Ministry of Public Health as well as believing in the capacity of the government to provide healthcare services. Interestingly, 70–80% of respondents comfortably expressed their trust toward the professional healthcare workers, yet only about half of the respondents believed professional healthcare workers can effectively deal with the COVID-19 which was a similar level of trust respondents placed in a capacity of the health system to effectively handle the crisis.

**Figure 1:**
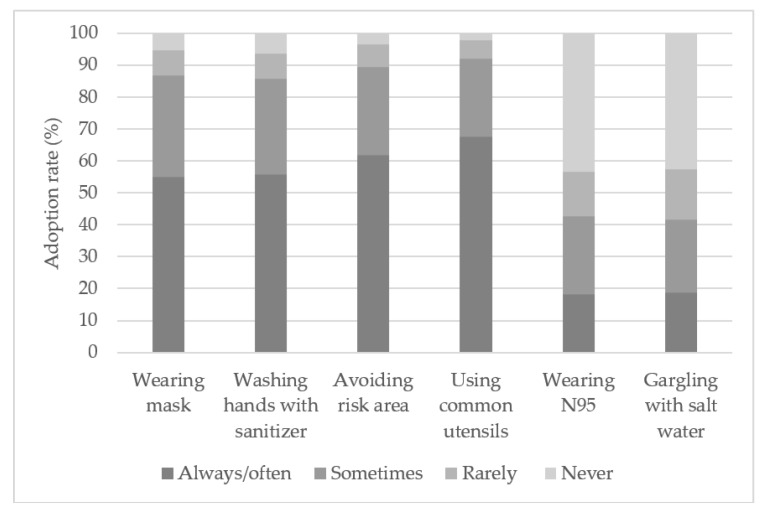
The prevalence of personal protective measures adoption against coronavirus disease 2019 (COVID-19). N95: A mask that filters at least 95% of airborne particles.

To further analyze the frequency of respondents adopting personal protective measures, Figure 1 illustrates that 95% of respondents reported having adopted personal protective measures as recommended by the authorities; 60% of respondents reported always practice the four personal protective measures while only 20% of respondents reported practicing the same for two other behaviors. It is clear that respondents were adopting the appropriate safety measures as recommended by health authorities. Despite only 60% of respondents reporting always adopting these safety measures during the early phase of the pandemic, these numbers rose to about 90% by the end of March [31].

Table 3 shows descriptive statistics and pairwise correlation coefficients. All presumed variables were significant and positive, and have preliminarily validated the relationship between public trust and policy compliance.

Since the elderly were found to have a higher risk of death from COVID-19, we further explored whether they were adopting these precautions less than the younger generation [44]. The Chi-square test reported that the elderly were 17 percentage points less likely to wear a mask when going out (71% vs. 88%, *p* < 0.01) than the younger group as shown in Table 4. Thus, during public health crises, the government and community should pay special attention to the elderly, either by promoting prevention knowledge or helping the elderly to have access to necessary personal protective equipment like surgical masks or cloth masks in time.

### 3.3. Hierarchical Regression Results 

After testing hierarchical regression, Table 5 illustrates a significant and positive relationship between public trust and respondents adopting personal protective measures. It also satisfies the condition of a mediator where an independent variable must be significantly associated with a mediating variable; a mediating variable must be significantly associated with a dependent variable; and an independent variable must be significantly associated with a dependent variable [45]. As shown in Model 4, trust in the government (β = 0.133, *p* < 0.01) was positively and significantly associated with the regularity of respondents adopting protections. As presented in Model 2, trust in the government was also positively and significantly associated with professional trust (β = 0.373, *p* < 0.001) and Model 5 further reveals a strong and significant relationship between professional trust and the frequency of practicing the precautions (β = 0.322, *p* < 0.001).

For the plausible confounders, it was found that married respondents (β = 0.183, *p* < 0.01), who resided in Bangkok (β = 0.104, *p* < 0.05), and those who felt worried about COVID-19 (β = 0.192, *p* < 0.001) were significantly and strongly associated with the frequency of respondents practicing these personal protective measures. Surprisingly, sex, age, education, and income had no impact when predicting the adoption of personal protective measures.

Model 6 in Table 5 reveals a certain impact of professional trust as being a mediating variable on the relationship between trust in the government and the adoption of precautions. As compared to Model 4, the β of trust in the government in Model 6 was reduced by 0.118 on the adoption of precautions, namely from (β = 0.133, *p* < 0.001) to (β = 0.015, *p* > 0.05). To test whether professional trust was a mediating variable between the relationship of trust in the government and the adoption of personal protective measures, we performed the bootstrapping test using the PROCESS procedure of SPSS and found that trust in the government has been 88.6% indirectly affected by the professional trust at a 95% bootstrapped confidence interval from 0.080 to 0.160. Essentially, this confirmed that professional trust was a full mediator between the relationship between public trust in the government and the willingness of respondents to comply with the safety measures introduced during COVID-19 outbreak in Thailand.

## 4. Discussion

The COVID-19 pandemic is the most deadly threat humankind has faced in the modern world while a vaccine was yet to be largely available, and one of the most effective means to mitigate the COVID-19 outbreak has been to appropriately protect oneself and comply with a control policy for collective safety. This study finds that the majority of Thai respondents adopted the newly introduced personal protective measures and only 5% indicated that they have never adopted any of these guidelines during the early phase of the pandemic. This corresponds to the findings that indicated 92% of respondents changed their social behavior even before the implementation of government policy in late March, whereby 94% practiced social distancing, 97% used personal protective equipment such as masks and 97% reported using sanitizer products [39]. Social determinants of marital status, residential area, and degree of worry were found to be associated with the prediction for the adoption rate of personal protective measures in Thailand whereas sex, age, education, and income were found to have no impact. However, when looking at each personal protective measure, the elderly were found to wear face masks less regularly than the younger generation.

This study contributes to the growing literature on public trust and policy compliance in several ways. Firstly, we found that public trust was positively related to the compliance of respondents practicing personal protective measures and reaffirmed that public trust in the government determines the tendency of policy compliance [1,2,3,4,5,6,7,19]. In Thailand, after the government established the Center for COVID-19 Situation Administration (CCSA) as a special task force responding to the pandemic when a strict lockdown policy was implemented in late March, immediate three-month financial compensation to those affected by the lockdown policy and vulnerable groups was also applied. The monetary assistance was particularly significant as it allowed the general public to adhere to the precautions and reduced emotional distress and psychological burdens in a time of crisis [46,47].

Secondly and more importantly, we assessed the mediating effect of professional trust and found that it fully mediated the relationship between public trust in the government and the adoption of personal protective measures. Although low public trust in the Thai government caused alarm, high professional trust has prompted the general public to comply with safety measures. As found in this study, professional trust has dual positive effects on health policy compliance, namely, a direct effect and a mediating effect on the relationship between trust in the government and policy compliance. During the crisis, professional trust served as a supplementary mechanism, empowering the government to increase the likelihood of citizens adopting personal protective measures. It is noticeable that the spokesperson for the CCSA is also a physician who has constantly reminded the general public to wear masks, wash hands often, avoid risk areas, and practice social distancing measures. As citizens placed their trust in physicians and perceived them as equipped with knowledge and compassion, they listened to their advice and tended to adopt these personal protective measures more frequently and this finally contributed to the successful effort in prevention and control of the pandemic. Indeed, knowledge, compassion, and the involvement of professional’s communities are keys to promote prevention and overcome epidemics [48,49,50,51]. However, healthcare professionals must be cautious not to influence the policy but remain grounded in their professional advice and fulfilled their fiduciary commitments; otherwise, their trust cannot be maintained [52].

Finally, since the COVID-19 pandemic is a global crisis that is closely related to all countries, shared experiences among countries are deemed crucial. Through preliminary outcome assessment of various countries in responding to this public health crisis, we reflected on the important role of public trust in the government and professional trust. When the level of trust in the government is low, professional trust fosters the adoption of personal protective measures and finally produces satisfactory outcomes as illustrated in Thailand. Likewise, in a country with traditionally high trust both in the government and professionals like Singapore, the pandemic is relatively contained. A country with high public trust but low professional trust can also bring the COVID-19 pandemic under control, as in China. In contrast, when the voice of healthcare professionals is being ignored in times of crisis, the consequences can be catastrophic, especially in a developing country with low trust in the government like Brazil which has currently reported the third-highest accumulated confirmed cases of COVID-19 worldwide [53].

### Limitations and Future Research

Our research is not without limitations. Firstly, this is a self-reported evaluation, and respondents might overestimate the frequency of practicing personal protective measures to be in line with the social norms. A subjective assessment of trust in the government is considered another possible bias since Thailand is infamous for a fragmented and fragile politics. Those who favor the current government might place higher trust in the government whereas the opposition might do just the opposite. Nonetheless, the capacity of the government to provide healthcare services is apolitical and, therefore, can represent a more objective appraisal of trust in government. Secondly, the socioeconomic characteristics of our respondents might not be representative, however, half of the respondents residing outside Bangkok was in line with the infected cases. Finally, the adoption of safety measures might be subject to social and cultural differences. A conclusion drawn from this study should be interpreted with caution and socio-cultural awareness.

Future research should further study other potential interactions between trust in the government and professional trust in relation to policy compliance in times of a public health crisis. The impact of rural and urban living on the relationship between trust and the tendency of policy compliance in times of crisis also needs further investigation. Finally, it would also be interesting to examine whether the mechanism found in this study is consistent in other diverse socio-cultural backgrounds.

## 5. Conclusions

Public trust is positively associated with the compliance of the general public to adopt personal protective measures during the unprecedented global public health crisis of the COVID-19 pandemic. More importantly, professional trust has a significant role in responding to the crisis and promoting policy compliance as the public strongly need to know how to accurately protect themselves from trusted sources. In a normal situation, professional trust plays a supporting role but in a time of crisis it plays a more prominent role. Francis Bacon once said, “Knowledge is power”; when coping with an unprecedented public health crisis that affected millions of lives such as the recent COVID-19 outbreak, knowledge and trust in professional healthcare workers is far more relevant. Meanwhile, the general public must also have a high degree of awareness and are willing to trust and follow these intervention guidelines. While the government still needs to increase public trust for future policy compliance, we propose that policymakers should consider the role of professional trust and wisely employ public trust simultaneously when designing future control interventions for a preventable disease like COVID-19. It is hoped that the finding from Thailand can provide a framework for other countries in promoting policy compliance, especially in times of uncertainty in countries with low public trust in the government.

## Figures and Tables

**Table 1:** Descriptive statistics of the demographic characteristics of the study population (*n* = 809).

Variable	Category	*n* (%)
Gender	Male	312 (38.6)
	Female	497 (61.4)
Age	<18	33 (4.1)
	18–29	214 (26.5)
	30–39	330 (40.8)
	40–59	212 (26.2)
	60 and above	20 (2.5)
Marital Status	Single	474 (58.6)
	Divorced	40 (4.9)
	Married	295 (36.5)
Education	Below college degree	206 (25.5)
	College degree and above	603 (74.5)
Residence	Bangkok	452 (55.9)
	Non-Bangkok	357 (44.1)
Personal Monthly Income (THB)	<5000 (very low)	66 (8.2)
	5000–15,000 (low income)	191 (31.8)
	15,000–30,000 (lower-middle)	247 (30.5)
	30,000–50,000 (upper-middle)	201 (24.8)
	>50,000 (high income)	104 (12.9)
Perceived vulnerability	Worried	591 (73.1)
	Affected	413 (51.1)

**Table 2:** Descriptive statistics for variables used in the study (*n* = 809).

Variable	Cronbach’s α	*n* (%)
Trust in the Government	0.82	
Trusts the central government		170 (21.0)
Trusts the local government		126 (15.6)
Trusts the Ministry of Public Health		392 (48.5)
Believes the government to have an effective policy		218 (26.9)
Believes health system to handle the crisis effectively		338 (41.8)
Believes health system to provide effective screening services		268 (33.1)
Believes health system to provide effective treatment services		368 (45.5)
Trust in Professional Healthcare Workers	0.85	
Trusts doctor		651 (80.5)
Trusts dentist		623 (77.0)
Trusts pharmacist		567 (70.1)
Trusts nurse		548 (67.7)
Believes healthcare workers to handle the crisis effectively		431 (53.3)
Compliance with the COVID-19 Control Measures	0.70	
Wears surgical/facial mask		765 (94.6)
Washes hands with an alcohol-based sanitizer		758 (93.7)
Avoids risk area		780 (96.4)
Uses common utensils when eating with others		792 (97.9)

**Table 3:** Descriptive statistics and Pearson correlation matrix.

Variable	Mean	SD	1	2	3	4	5	6	7	8	9	10
1. Measures’ compliance	3.67	0.75	1									
2. Trust in the government	3.06	0.68	0.07 *	1								
3. Professional trust	3.79	0.63	0.25 **	0.39 **	1							
4. Male	0.39	0.49	−0.04	0.09 **	0.06	1						
5. Age	2.97	0.89	0.02	0.04	0.03	0.01	1					
6. Married	0.36	0.48	0.09 **	0.01	−0.04	0.01	0.38 **	1				
7. Education	0.75	0.44	0.07	−0.06	0.10 **	−0.14 **	0.16 **	−0.02	1			
8. Income	3.11	1.15	0.05	−0.09 *	0.09 *	-0.06	0.43 **	0.18 **	0.50 **	1		
9. Bangkok	0.56	0.50	0.07 *	0.00	-0.06	0.04	0.04	−0.01	−0.01	0.13 **	1	
10. Worry	4.01	0.91	0.22 **	−0.17 **	-0.03	−0.11 **	−0.05	−0.01	0.05	0.02	0.10 **	1

Notes: *n* = 809; * *p* < 0.05, ** *p* < 0.01. SD: Standard deviation; 1–10: 1 = Measures’ compliance, 2 = Trust in the government, 3 = Professional trust, 4 = Male, 5 = Age, 6 = Married, 7 = Education, 8 = Income, 9 = Bangkok, 10 = Worry.

**Table 4:** A cross-tabulation analysis of two different age groups in wearing masks.

Group	Wearing Mask	Not Wearing Mask
Elderly group	71.4% (25)	28.6% (10)
Non-elderly group	87.5% (677)	12.5% (97)
Count	86.8% (702)	13.2% (107)

Notes: *n* = 809; Pearson chi-square test is 15.967, *p* < 0.01.

**Table 5:** Ordinary least squares (OLS) hierarchical regressions analyzing the relationship between public trust in government, professional trust, and the adoption of personal protective measures.

Variable	Professional Trust		Adoption of Personal Protective Measures			
	Model 1	Model 2 (Model 1 + Trust in the Government)	Model 3	Model 4 (Model 3 + Trust in Government)	Model 5 (Model 3 + Professional Trust)	Model 6 (Model 3 + Trust in the Government + Professional Trust)
Control variables						
Male	0.098 * (0.046)	0.063 (0.042)	−0.020 (0.053)	−0.032 (0.053)	−0.051 (0.052)	−0.052 (0.052)
Age	0.005 (0.029)	−0.020 (0.027)	−0.013 (0.034)	−0.022 (0.034)	−0.014 (0.033)	−0.015 (0.033)
Married	−0.068 (0.049)	−0.069 (0.045)	0.162 ** (0.058)	0.161 ** (0.057)	0.183 ** (0.056)	0.183 ** (0.056)
Education	0.107 (0.059)	0.100 (0.054)	0.106 (0.069)	0.104 (0.069)	0.072 (0.067)	0.072 (0.067)
Income	0.038 (0.025)	0.067 ** (0.023)	−0.001 (0.029)	0.009 (0.029)	−0.013 (0.028)	−0.012 (0.028)
Bangkok	−0.089 * (0.045)	−0.101 * (0.041)	0.076 (0.053)	0.072 (0.052)	0.105 * (0.051)	0.104 * (0.051)
Worry	−0.016 (0.024)	0.029 (0.022)	0.176 *** (0.028)	0.192 *** (0.029)	0.182 *** (0.027)	0.183 *** (0.028)
Predictors						
Trust in the government		0.373 *** (0.030)		0.133 ** (0.038)		0.015 (0.040)
Professional trust					0.322 *** (0.040)	0.316 *** (0.044)
Constant	3.678 *** (0.133)	2.364 *** (0.161)	2.836 *** (0.156)	2.368 *** (0.205)	1.651 *** (0.210)	1.622 *** (0.224)
F value	2.982 **	22.501 ***	8.041 ***	8.654 ***	15.762 ***	14.011 ***
R^2^	0.025	0.184	0.066	0.080	0.136	0.136

Notes: *n* = 809; * *p* < 0.05, ** *p* < 0.01, *** *p* < 0.001.

## Data Availability

The data analyzed in this study are available on reasonable request to the corresponding author.
